# A Novel Secretion Pathway of *Salmonella enterica* Acts as an Antivirulence Modulator during Salmonellosis

**DOI:** 10.1371/journal.ppat.1000036

**Published:** 2008-04-04

**Authors:** Ohad Gal-Mor, Deanna L. Gibson, Dan Baluta, Bruce A. Vallance, B. Brett Finlay

**Affiliations:** 1 Michael Smith Laboratories, University of British Columbia, Vancouver, British Columbia, Canada; 2 Division of Gastroenterology, University of British Columbia and BC Children's Hospital, Vancouver, British Columbia, Canada; Pasteur Institute, France

## Abstract

*Salmonella* spp. are Gram-negative enteropathogenic bacteria that infect a variety of vertebrate hosts. Like any other living organism, protein secretion is a fundamental process essential for various aspects of *Salmonella* biology. Herein we report the identification and characterization of a horizontally acquired, autonomous and previously unreported secretion pathway. In *Salmonella enterica* serovar Typhimurium, this novel secretion pathway is encoded by STM1669 and STM1668, designated *zirT* and *zirS*, respectively. We show that ZirT is localized to the bacterial outer membrane, expected to adopt a compact β-barrel conformation, and functions as a translocator for ZirS. ZirS is an exoprotein, which is secreted into the extracellular environment in a ZirT-dependent manner. The ZirTS secretion pathway was found to share several important features with two-partner secretion (TPS) systems and members of the intimin/invasin family of adhesions. We show that *zirTS* expression is affected by zinc; and that in vivo, induction of *zirT* occurs distinctively in *Salmonella* colonizing the small intestine, but not in systemic sites. Additionally, strong expression of *zirT* takes place in *Salmonella* shed in fecal pellets during acute and persistent infections of mice. Inactivation of ZirTS results in a hypervirulence phenotype of *Salmonella* during oral infection of mice. Cumulatively, these results indicate that the ZirTS pathway plays a unique role as an antivirulence modulator during systemic disease and is involved in fine-tuning a host–pathogen balance during salmonellosis.

## Author Summary

Bacteria of the *Salmonella* genus are important human pathogens and a leading cause of food-borne illness. Like for all other living organisms, protein secretion is a fundamental process, which is required for many different aspects of *Salmonella* biology including biogenesis of organelles, nutrient acquisition, and virulence. In this work we describe a new secretion pathway in *Salmonella* termed ZirTS. This pathway consists of an exported protein (ZirS) and a designated membrane translocator (ZirT), which mediates the secretion of ZirS to the extracellular milieu. Using a mouse model of *Salmonella* infection, we found that the ZirTS system is induced in *Salmonella* colonizing the small intestine and in *Salmonella* shed in fecal pellets during acute and persistent infections. Interestingly, inactivation of ZirTS results in a hypervirulence phenotype of *Salmonella* during oral infection of mice. These observations indicate that the ZirTS pathway plays a unique role as an antivirulence modulator and is involved in fine-tuning host–pathogen interactions during disease. Our study elucidates an emerging theme in pathogenesis emphasizing the importance of pathogens to limit their effects upon the cells they infect in order to achieve a balance with their host.

## Introduction

*Salmonella* spp. are Gram-negative enteropathogenic bacteria that infect a variety of mammalian, avian and reptile hosts. Infection by this highly versatile pathogen can lead to different outcomes including asymptomatic carriage, gastroenteritis, or severe, life-threatening systemic disease, known as typhoid fever. The nature and the severity of the disease depend upon the serovar of the infecting *Salmonella* as well as the species and immunological status of the infected host [1].

The two hallmarks of *Salmonella enterica* serovar Typhimurium (*S.* Typhimurium) pathogenesis are the invasion of non-phagocytic cells such as epithelial cells of the intestinal mucosa, and the survival and replication inside infected phagocytic cells. Both mechanisms, as well as many of the virulence determinants used by *S.* Typhimurium, are directly linked to genes encoded within large horizontally acquired regions of the chromosome termed *Salmonella* pathogenicity islands.

Protein secretion is a ubiquitous cellular function found in organisms of all kingdoms. Gram-negative bacteria secrete a wide range of proteins whose functions include biogenesis of organelles, nutrient acquisition, virulence, efflux of toxins, and injection of virulence factors (effectors) into host cells. Protein export from the bacterial cytoplasm to the surface or the extracellular milieu requires transport across the inner membrane (IM), periplasm, and outer membrane (OM) of the cell envelope. In Gram-negative bacteria, several secretion pathways have evolved to fulfill this task [2],[3]. The auto-transporters (ATs) and the two-partner secretion (TPS) systems (often classified as the Type V Secretion System) have been the focus of much interest in recent years due to their prime role in virulence traits of Gram-negative pathogens [4],[5].

ATs are single functional units consisting of modular domains including: an N-terminal signal sequence that targets the protein to the general secretion (Sec) machinery at the IM; the passenger domain, which harbors the specific effector function; and the C-terminal translocation unit that forms, once inserted into the OM, a β-barrel secondary structure that mediates the secretion of the passenger domain. ATs are synthesized as pre-pro-proteins, and after cleavage of the signal peptide, the pro-protein is released into the periplasm. The passenger domain is then exported through the OM via the translocation unit, often cleaved off and released into the extracellular milieu [6],[7].

In contrast to the ATs, which are synthesized as a single polypeptide, in TPS systems the passenger domain and the transporter domain are translated as two separate proteins, referred to by the generic terms TpsA and TpsB, respectively [8]. TpsA proteins are synthesized with an N-terminal cleavable signal peptide and transported across the IM by the Sec machinery. Subsequently, TpsA substrates transit through the periplasmic space to their cognate secretion partner (TpsB) which then facilitates their secretion [9]. The TpsB cluster members show characteristic features of integral OM proteins and like TpsA, are thought to be exported across the IM by the Sec apparatus [9]. Conserved amphipathic motifs throughout their sequence indicate that TpsB proteins are likely to contain high numbers of transmembrane β-strands [10]. This secondary structure is believed to adopt a β-barrel conformation forming a pore in the OM that enables the translocation of TpsA across the OM into the extracellular environment.

Another group of proteins, which are conceptually analogous to ATs is the intimin/invasin (Int/Inv) family of adhesins. These family members are specialized OM proteins found in strains of *Yersinia* spp. (Inv), pathogenic *E. coli* (Int), and *Citrobacter* spp. (Int) that mediate adhesion of these pathogens to their hosts. Both invasins and intimins are translocated from the cytoplasm across the IM via the Sec-translocase and are related to each other both in terms of sequence and structure. The structure of Int/Inv includes a C-terminal C-type lectin receptor-binding domain, which is separated from a membrane-embedded N-terminal domain by several tandem Ig-like repeats, four in invasin and three in intimin. The conserved N-terminus domain is believed to form a β-barrel in the OM, which is used for the export of the C-terminal region. The extracellular C-terminus of Int/Inv is responsible for the receptor binding (Tir and β1 integrin, respectively) [11],[12]. Not much is known about the secretion mechanisms of Int/Inv, but based on existing similarities with ATs [13], it has been proposed that Int/Inv are secreted by an ATs-like mechanism [14].

In this report we describe the identification and characterization of a novel secretion pathway in *Salmonella*, named ZirTS. We show that ZirTS share important characteristics with the TPS systems and the Int/Inv family, and demonstrate that ZirTS play a unique role as an antivirulence modulator during systemic disease in mice.

## Results

### Identification of *zirTS* in a conserved *Salmonella* genomic island

Many virulence factors are pathogen-specific, however, a growing group of identified virulence determinants has been shown to harbor homology to various eukaryotic proteins or domains [15],[16], presumably as a result of continuous co-evolution with the eukaryotic host. Based on this idea, we developed a bioinformatic screen aimed at identifying *Salmonella* open reading frames (ORFs) that: (1) are absent from related non-pathogenic bacterial genomes and; (2) possess homology to known eukaryotic domains.

Screening the *Salmonella* Typhimurium LT2 genome while applying these bioinformatic filters led to the identification of an unknown ORF designated STM1668, located 26-bp downstream to an ‘invasin-like’ annotated gene (STM1669). Herein we rename STM1668 and STM1669 *zirS* and *zirT,* respectively, (see below).

No homologs of ZirS were found in the currently available genome databases in any bacterial genome outside of the *Salmonella* genus; however, weak homology was found to several eukaryotic proteins including a human zinc finger protein (NP_065798, 24% identity and 39% similarity over 199 amino acids). Additionally, *zirS* was found to be A+T rich (59.7%) in comparison to the rest of the *S.* Typhimurium genome (47%) and was located within a previously identified genomic island, GEI 1664/1678 [17]. These observations indicate that the *zirS* region was most likely acquired by a lateral gene transfer event during the evolution of *Salmonella*.

**Figure 1:**
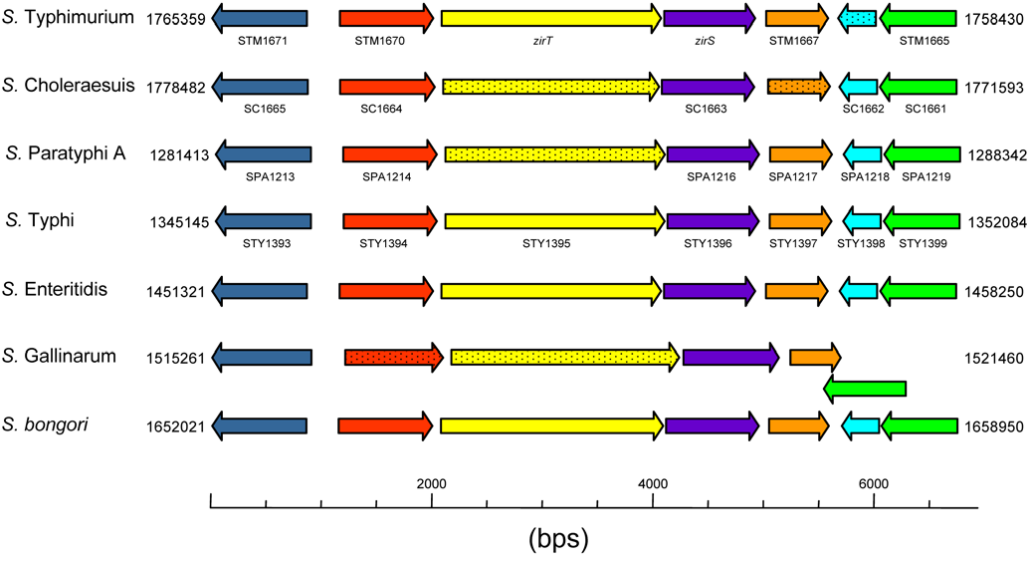
*zirS* and *zirT* are organized in a conserved *Salmonella* genomic island. The *zirTS* corresponding region was compared between different *Salmonella* species and serovars, whose genome sequence is currently available, including: *S. enterica* serovar Typhimurium LT2; *S. enterica* serovar Choleraesuis str. SC-B67; *S. enterica* serovar Paratyphi A str. ATCC 9150; *S. enterica* serovar Typhi str. CT18; *S. enterica* serovar Enteritidis PT4 NCTC 13349; *S. enterica* serovar Gallinarum 287/91 NCTC 13346; and *S. bongori* 12419 ATCC 43975. Spotted arrows indicate possible frameshift mutations. ORF annotations (when available) and chromosomal position of the coresponding regions are indicated.

Interestingly, a highly conserved organization of the *zirS* region was found in all of the available *Salmonella* serovar genome sequences including *S. bongori* (Figure 1), implying that the lateral transfer event occurred before the divergence of *S. enterica* from the species *S. bongori* (∼35 to 40 million years ago), but after the split of *Salmonella* from the genus *Escherichia* (∼120 to 160 million years ago) [18],[19].

The neighboring ORFs adjacent to *zirT* and *zirS* include STM1670 and STM1667. STM1670 is annotated as a putative serine/threonine protein kinase and located 74-bp upstream to *zirT*. STM1670 homologs are currently found only in *Salmonella* databases, suggesting that it might be a unique *Salmonella* protein. STM1667, which is encoded 98-bp downstream to *zirS* contains a conserved peroxiredoxin domain and is annotated as a putative thiol peroxidase.

### ZirS is secreted into the extracellular milieu in a Sec-dependent manner

**Figure 2:**
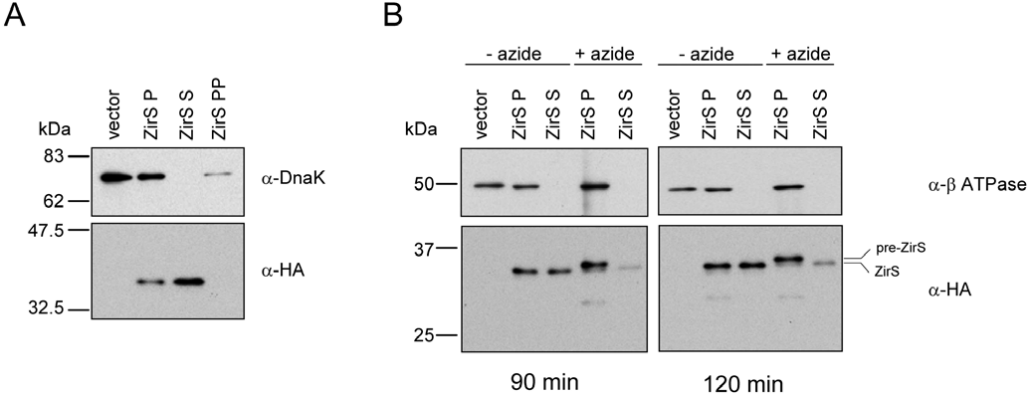
ZirS is an exoprotein secreted in a Sec-dependent manner. A. ZirS is secreted into the extracellular environment. *S.* Typhimurium strains harboring ZirS-HA in the presence of *zirT* (pOG-zirTS-HA; ZirS) or the empty vector (pWSK29; vector) were grown in LB to late-logarithmic phase. Whole bacterial cell pellets (P), culture media supernatant (S), and the periplasmic fraction (PP) were analyzed by Western-blot using an anti-HA antibody (bottom panel). To show that the ZirS-HA detected in the supernatant is not a result of cells lysis, the same blot was reprobed for the cytoplasmic protein, DnaK (top panel). B. ZirS is secreted in a Sec translocase-dependent manner. *S.* Typhimurium harboring pOG-zirTS-HA (ZirS) or the empty vector (vector) were grown in LB to late exponential phase, washed and incubated for additional 90 or 120 min in fresh LB supplemented with (+) or lacking (−) 2 mM sodium azide. The cellular and the secreted levels of ZirS were evaluated in the whole bacterial cell pellets (P) and the secreted fractions (S) by Western-blot using an anti-HA antibody (bottom panel). As a fractionation control, the blot was probed against the β-ATPase membrane protein (top panel). Protein samples were normalized according to the optical density (O.D._600_) of the cultures and separated on SDS-13.5% PAGE.

ZirS is predicted to be a 276 amino acid protein with an estimated molecular mass of 30.8 kDa. Sequence analysis of ZirS using the SignalP 3.0 program [20] (http://www.cbs.dtu.dk/services/SignalP/) predicted a typical prokaryotic Sec-dependent signal sequence at its N-proximal region, with a potential cleavage site between amino acids 24 and 25 (VLA^▾^DS). In Gram-negative bacteria, the presence of a signal sequence suggests that the protein is processed and exported across the IM in a Sec-dependent fashion. This process involves the cleavage of the signal peptide by a LepB leader peptidase (type I signal peptidase) and requires ATP hydrolysis by a designated ATPase, which provides the driving force for translocation (reviewed in [21]). To examine this hypothesis experimentally, a tagged version of ZirS was constructed using a C-terminal two-hemagglutinin (2HA) tag and cloned into a low copy number vector in the presence of the upstream gene, *zirT* (pOG-zirTS-HA). As illustrated in Figure 2A, expression of *zirS*-HA (in the presence of *zirT*) in *S.* Typhimurium led to prominent secretion of ZirS-HA into the medium as detected by Western-blot analysis.

To assess the contribution of the Sec-translocon to the extracellular export of ZirS, the secretion of ZirS-HA in the absence and presence of azide was studied. Low concentrations of azide (2 mM) specifically inhibit SecA, the ATPase component of the Sec-complex, and therefore interfere with Sec-dependent protein secretion, resulting in accumulation of pre-proteins in the cytoplasm [22],[23]. In performing this experiment, *Salmonella* strain expressing ZirS-HA (pOG-zirTS-HA) that was grown in LB to late logarithmic phase was washed, resuspended in fresh medium, and incubated for 90 or 120 min in the presence or absence of 2 mM sodium azide. Subsequently, the intracellular and the secreted ZirS-HA were analyzed by Western-blot. As demonstrated in Figure 2B, the presence of low concentrations of azide strongly reduced the secretion of ZirS-HA into the medium and led to accumulation of a higher molecular-weight (pre-ZirS-HA) isoform in the cytoplasm. We concluded from these experiments that the secretion of ZirS into the extracellular environment is dependent on the function of the Sec-translocon and involved signal peptide cleavage at the N-terminus of ZirS.

### ZirT is homologous to members of the Int/Inv family of adhesins

**Figure 3:**
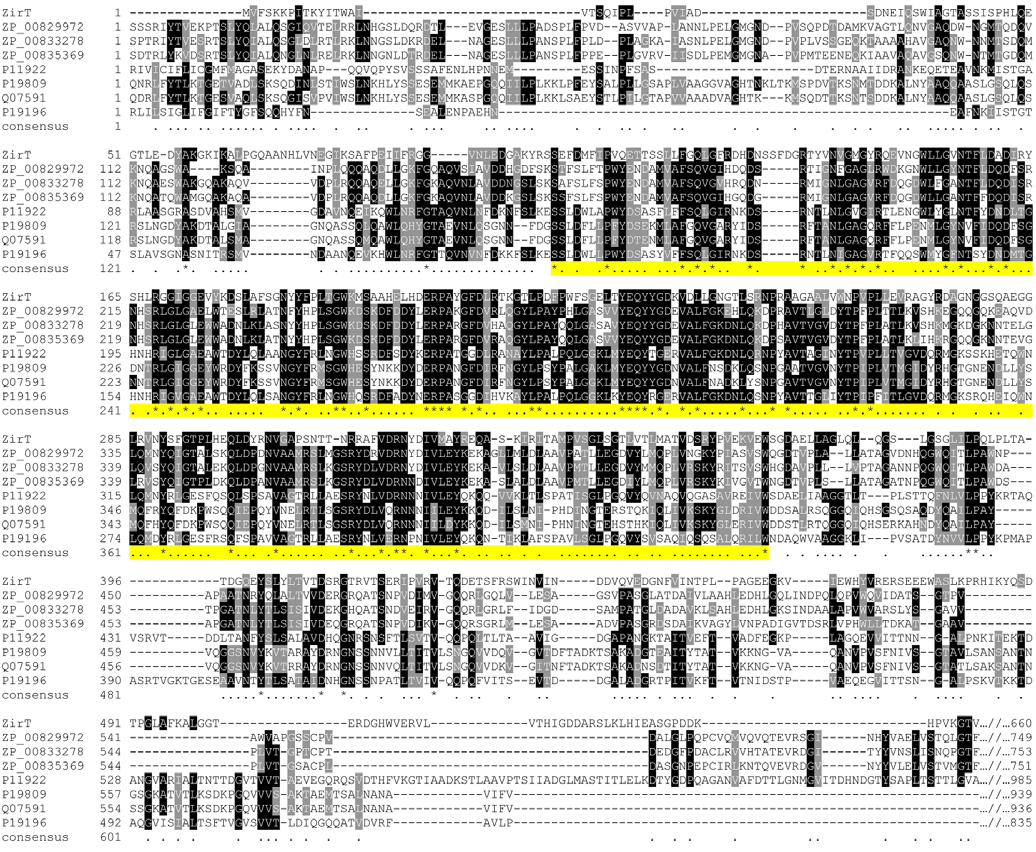
ZirT is homologous to the Int/Inv family members. Amino acid sequence alignment of ZirT (NP_460627); *Yersinia frederiksenii* predicted invasin (ZP_00829972); *Yersinia intermedia* predicted invasins (ZP_00833278 and ZP_00835369); *Yersinia pseudotuberculosis* invasin (P11922); *Escherichia coli* O127:H6 intimin (P19809); *Citrobacter rodentium* intimin (Q07591); and *Yersinia enterocolitica* invasin (P19196) is presented. Sequence alignment was performed by ClustalW and the output was reformatted by BoxShade 3.21. Amino acid identity is shown in black and similar amino acids are shown in gray. The highly similar region spanning amino acids 88 to 368 of ZirT is highlighted.

ZirT, encoded by the gene immediately upstream to *zirS*, is predicted to be a 660 amino acid protein with an estimated molecular mass of 72.7 kDa. In contrast to ZirS that showed no prokaryotic homologs outside of the genus *Salmonella*, a bioinformatic search against non-redundant protein databases revealed several bacterial protein groups that share significant homology with ZirT. All are known OM proteins including various invasins and intimins from different Gram-negative pathogens (Figure 3). More precise comparison of ZirT to these proteins revealed that the sequence similarity is concentrated within the mid-N-terminal region of ZirT, spanning from amino acid 88 to 368. Importantly, these homologous regions are thought to form porin-like β-barrels in the bacterial OM [13].

### ZirT is folded into a β-barrel protein in the outer-membrane

**Figure 4:**
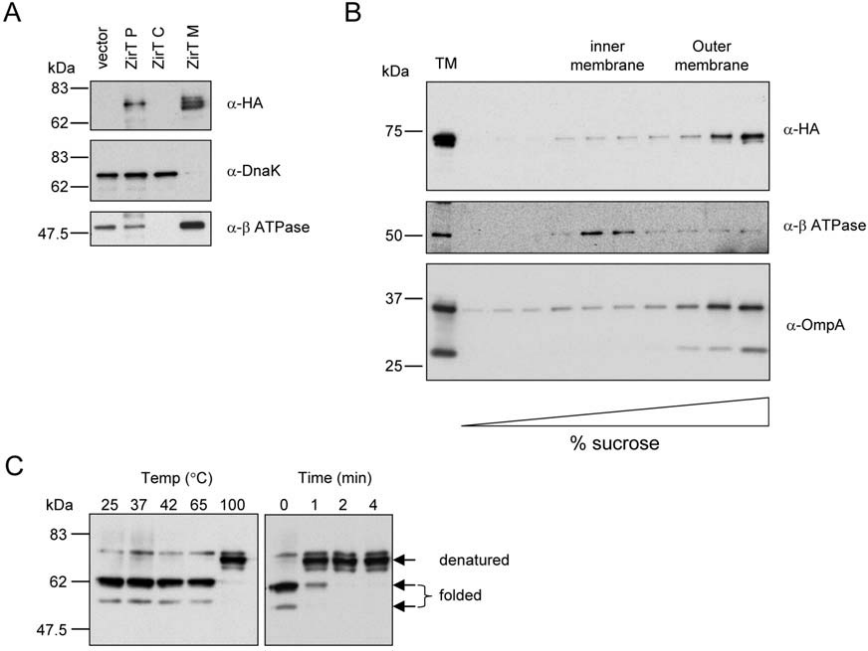
ZirT is arranged into a β-barrel structure in the outer membrane. A. ZirT is localized into the cell envelope. *S.* Typhimurium SL1344 expressing ZirT-HA was grown in LB to late-logarithmic phase followed by cellular fractionation as explained in the Materials and Methods section. Proteins from the whole bacterial cell pellets (P), cytoplasmic fraction (C), and membranes (M) were analyzed by SDS–10% PAGE and immunoblotted with anti-HA. As a control for cytoplasmic proteins, the blot was probed with anti-DnaK. Antisera raised against the β-subunit of ATPase were used as a control for membrane proteins. B. ZirT is an outer membrane protein. Total membranes fraction (TM) was isolated from SL1344 expressing ZirT-HA by ultracentrifugation, applied to the top of a sucrose density gradient and subjected to ultracentrifugation at 100,000 *g* for 16 h. Fractions were collected from the bottom of the gradient and aliquots were separated on SDS-10% PAGE followed by Western blotting. As controls for OM and IM proteins we used polyclonal antisera raised against OmpA (two OmpA isoforms are shown) and the β-subunit of ATPase, respectively. C. ZirT is expected to adopt a compact β-barrel secondary structure. Total protein extract from whole cell lysate of *S.* Typhimurium expressing ZirT-HA was subjected to heat-modifiable electrophoretic mobility analysis. Equal protein portions were heated for 10 min at 25, 37, 42, 65, and 100°C (left panel) or boiled for 0, 1, 2 or 4 min (right panel), immediately placed on ice and separated in SDS-8% PAGE followed by Western blotting using an anti-HA antibody. The ZirT-HA bands representing the folded and the denatured forms are indicated.

Further sequence analysis of ZirT also predicted a Sec-dependent signal sequence in the N-terminus with a potential cleavage site between amino acids 27 and 28 (VIA^▾^DS), which supported the possible export of ZirT from the cell cytoplasm. In agreement with the sequence homology found, other localization prediction tools (PSORTb v.2.0 http://www.psort.org/) [24] suggested a subcellular localization of ZirT in the OM, with 31 predicted trans-membrane β-segments (TMBETA-NET, http://psfs.cbrc.jp/tmbeta-net/) [25]. In order to investigate the subcellular localization of ZirT, a C-terminus HA tagged version was constructed and cellular fractionation analysis of *Salmonella* cells expressing ZirT-HA (pOG-zirT-HA) was performed. This experimental approach showed the localization of ZirT-HA in the cellular membranes fraction (Figure 4A).

To further characterize the precise localization of ZirT in the cell envelope, we utilized sucrose density gradient ultracentrifugation fractionation. *S*. Typhimurium total membranes fraction was isolated and subjected to ultracentrifugation through a sucrose density gradient (30–60% sucrose, w/v). The specific localization of ZirT was determined based on the presence of OmpA and β-ATPase used as controls for the OM and the IM fractions, respectively [26],[27]. As shown in Figure 4B, ZirT-HA was found to be distinctively localized into the OM fractions.

In general, many OM proteins with β-barrel structures exhibit heat-modifiable electrophoretic mobility behavior, in which strong resistance to denaturation in the presence of 1% SDS is observed, unless heated to 100°C. Consequently, the folded and the compact β-barrel conformations migrate more quickly in SDS-PAGE than their denaturated forms [28]. As ZirT was found to have sequence similarity to known β-barrel OM proteins and predicted to be amphipathic β-strand rich, we investigated whether ZirT also exhibited heat-modifiable electrophoretic mobility. When protein extracts from *Salmonella* cells expressing ZirT-HA (pOG-zirT-HA) were incubated in a sample buffer without boiling and analyzed by SDS-PAGE, instead of running at its expected denatured position of ∼75 kDa, non-boiled ZirT-HA migrated mainly as a faster protein band at ∼62 kDa (and a secondary band at ∼55 kDa). Heat denaturation of the samples before analysis reproduced the unfolded form in a time and temperature-dependent manner (Figure 4C). These data suggest that the folded, mature ZirT is arranged into β-barrel architecture in the OM.

It is noteworthy that Western-blot analyses against the denatured tagged version of ZirT allowed the detection of 2–3 distinct molecular-weight bands (Figure 4). This observation might indicate possible processing of ZirT, resulting in different protein isoforms.

### ZirS and ZirT compose a novel secretion system in *Salmonella*

**Figure 5:**
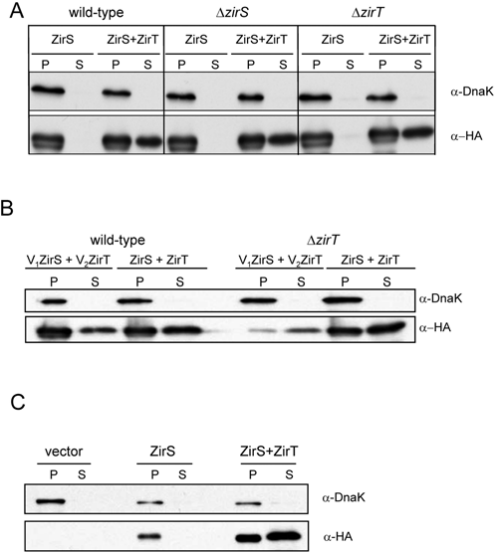
ZirS and ZirT compose an autonomous secretion system in *Salmonella.* A. The secretion of ZirS is ZirT-dependent. Wild-type *S.* Typhimurium (wild-type) or its isogenic strains harboring a deletion mutation in *zirS* (Δ*zirS*) or *zirT* (Δ*zirT*) expressing ZirS-HA only (pOG-zirS-HA; ZirS) or both ZirT and ZirS-HA (pOG-zirTS-HA; ZirS+ZirT) were grown in LB to late-logarithmic phase. To evaluate the expression and the secretion of ZirS-HA, whole bacterial cell pellets (P) and culture media supernatant (S) were analyzed by immunoblot using antibodies against hemagglutinin (bottom panel). To show that the ZirS-HA detected in the supernatant is not a result of bacterial cell lysis and that equal amounts of protein were loaded, the same blot was reprobed for the bacterial cytoplasmic protein, DnaK (top panel). B. Complementation of ZirS secretion in the presence of ZirT in *cis* and *trans*. The secretion of ZirS-HA was analyzed in wild-type *S.* Typhimurium (wild-type) or in a *zirT* mutant strain (Δ*zirT*) expressing ZirT and ZirS-HA either in *cis* from a single low copy number construct (pOG-zirTS-HA; ZirS+ZirT) or in *trans*, from two different, but similar-copy numbered vectors (pOG-zirS-HA and pOG-zirT-4; V_1_ZirS+V_2_ZirT). Whole bacterial cell pellets (P) and the secreted fractions (S) were analyzed by Western-blot using antibodies against HA (bottom panel) or DnaK (top panel). C. Expression of ZirS and ZirT in a heterologous host leads to the secretion of ZirS to the extracellular milieu. *E. coli* DH5α harboring an empty vector (vector), ZirS-HA only (pOG-zirS-HA; ZirS), or ZirS-HA and ZirT (pOG-zirTS-HA; ZirS+ZirT) were grown in LB. Whole bacterial cell pellets (P) and the secreted fractions (S) were analyzed by Western-blot as outlined above.

We next focused our interest on examining possible interactions between ZirT and ZirS. The secretion of ZirS-HA was, therefore, analyzed while expressed from a low-copy number construct harboring ZirS-HA alone (pOG-zirS-HA), or from a plasmid containing both ZirT and ZirS-HA (pOG-zirTS-HA). This assessment was done in three different *S.* Typhimurium genetic backgrounds (wild-type, Δ*zirS*, and Δ*zirT* strains) using immunoblots against the cellular and the secreted protein fractions. Interestingly, expression of ZirS-HA was observed in both the presence and absence of ZirT, as evidenced in the cellular fractions. However, in contrast to protein expression, secretion of ZirS-HA was only detected when ZirT was co-expressed (Figure 5A). Thus, secretion, but not expression, of ZirS was found to be dependent on the presence of ZirT.

Unexpectedly, the same results were observed in all three genetic backgrounds, including the wild-type and a Δ*zirS* mutant strain, both carrying a chromosomal copy of *zirT* that did not seem to support the secretion of ZirS-HA expressed from an episomal construct. Two possible interpretations of this result were: (1) the nature of the interaction between ZirS and ZirT requires specific stoichiometry that was not achieved from chromosomal expression of ZirT, or that (2) the secretion of ZirS demands the presence of ZirT in *cis*. To examine these possibilities we analyzed the expression and the secretion of ZirS-HA in the presence of ZirT that was provided either in *cis* from the same episomal construct (pOG-zirTS-HA) or in *trans* from a different vector with a similar (low) copy-number (pOG-zirS-HA and pOG-zirT-4). As can be seen in Figure 5B, providing ZirT either in *cis* or in *trans* from a similar copy number vector was able to complement the secretion of ZirS, implying that particular stoichiometry of ZirS and ZirT might be required for efficient secretion of ZirS.

To gain further insight into the nature of ZirS secretion, we expressed the HA-tagged version of ZirS alone (pOG-zirS-HA) or together with ZirT (pOG-zirTS-HA), in a heterologous *E. coli* K-12 host that does not possess any homologs of ZirTS (or any neighboring genes). Introducing ZirS-HA alone into an *E. coli* host resulted in detectable expression, but not secretion of ZirS-HA. In contrast, introducing ZirS-HA together with ZirT led to prominent secretion of ZirS-HA into the medium by *E. coli* (Figure 5C). We concluded from this experiment that *zirTS* encodes an autonomous and self-sufficient secretion system, in which ZirS is secreted in a strict ZirT-dependent manner.

Cumulatively, the data presented describing the nature of ZirT and ZirS are consistent with several key characteristics of TPS systems and/or the Int/Inv family. These similarities includes: (1) primary sequence homology to various intimins and invasins; (2) ZirT being an outer membrane β-barrel protein, similar to TpsB or the N-terminus module of the Int/Inv members; (3) like TpsA, ZirS seems to be translocated from the cytoplasm across the IM via the Sec translocase; (4) ZirT containing a prototypical N-terminal signal sequence, as the TpsB and the Int/Inv members; and (5) ZirS being secreted into the extracellular milieu in an explicit ZirT-dependent manner, analogous to the relation between TpsA and TpsB.

Nevertheless, despite the shared similarities, some fundamental differences exist between the ZirTS and the compared systems (see Discussion). Based on this, we suggest that the ZirTS secretion system is functionally similar to the TPS pathway, but represents a distinctive secretion pathway in *Salmonella*.

### The transcription factor OxyR and zinc are involved in *zirTS* regulation

**Figure 6:**
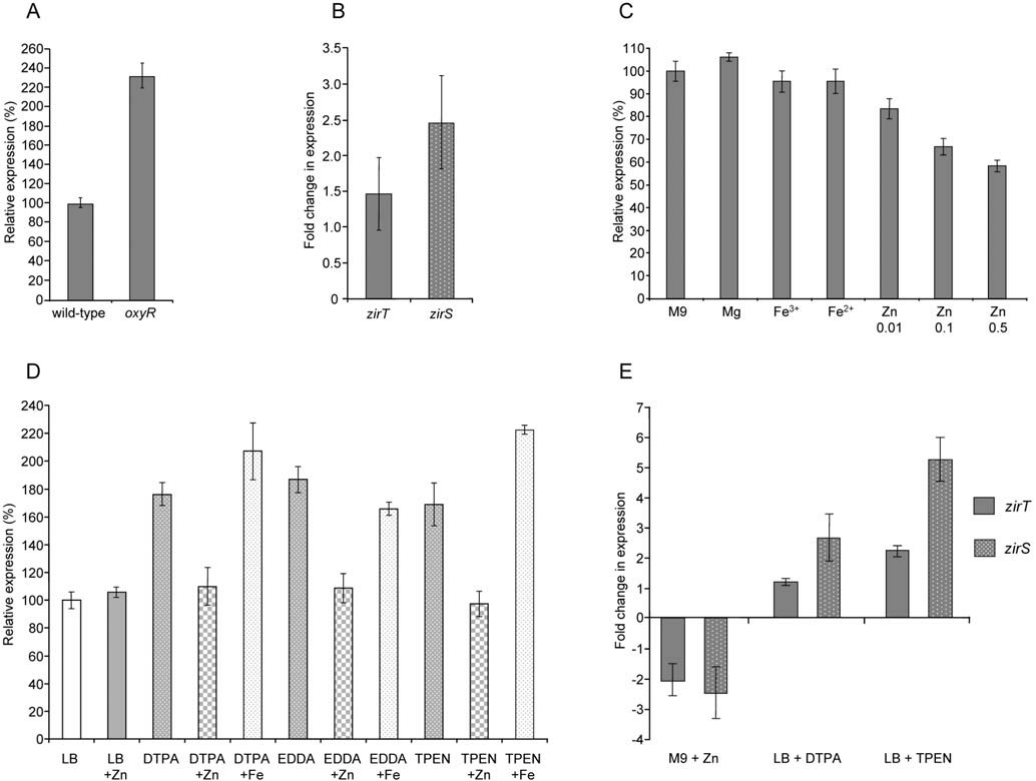
The ZirTS pathway is negatively regulated by zinc and OxyR. A. OxyR negatively regulates the expression of *zirTS*. Wild-type *S.* Typhimurium SL1344 (wild-type) and an isogenic strain harboring a mutation in *oxyR* (*oxyR*) expressing *zirTS::lacZ* were grown in M9 minimal medium to late stationary phase. β-galactosidase assay was performed to evaluate the expression of *zirTS::lacZ* in each strain. The presented values represent the average of at least 7 independent cultures with a standard deviation shown by the error bars. B. Expression of *zirT* and *zirS* in the *oxyR* background vs. the wild-type strain as determined by q-RT-PCR. RNA was harvested from SL1344 and the *oxyR* mutant strains grown in M9; reverse-transcribed and the expression of *zirT* and *zirS* was examined by quantitative real-time PCR. The fold change in the expression of *zirT* and *zirS* in the *oxyR* background, relative to their expression in the wild-type strain is presented. Expression was normalized using the housekeeping *rpoD* gene as a control. The results represent the average of 4 independent experiments (each included 3–5 replicates) with a standard deviation shown by the error bars. C. Expression of *zirTS* in the presence of metals. *S.* Typhimurium SL1344 expressing *zirTS::lacZ* was grown in M9 medium (M9) or M9 supplemented with the following metal salts: 0.1 mM MgSO_4_ (Mg), 0.1 mM FeCl_3_ (Fe^3+^), 0.1 mM FeSO_4_ (Fe^2+^), and ZnSO_4_ (Zn) in the indicated concentrations (0.01, 0.1, or 0.5 mM). Cultures were grown to a late stationary phase and β-galactosidase assay was performed to evaluate the expression levels of *zirTS::lacZ* under each condition. The presented values represent the average of at least 8 independent cultures with a standard deviation shown by the error bars. D. Expression in the presence of metal chelators. *S.* Typhimurium SL1344 expressing *zirTS::lacZ* was grown in LB broth (LB) or LB supplemented with the following compounds: 1 mM ZnSO_4_ (LB+Zn), 0.1 mM metal chelators (DTPA, EDDA, or TPEN), metals chelators with 1 mM ZnSO_4_ (Zn), or metal chelators with 1 mM FeSO_4_ (Fe). Cultures were grown to a late stationary phase following by β-galactosidase assay. The presented values represent the average of at least 8 independent cultures with a standard deviation shown by the error bars. E. Expression of *zirT* and *zirS* in the presence and absence of zinc and metal chelators as determined by q-RT-PCR. RNA was harvested from wild-type *Salmonella* cultures that were grown either in M9 medium in the presence or absence of 0.5 mM ZnSO_4_; or in LB supplemented with or lacking DTPA (0.1 mM) or TPEN (0.05 mM). RNA was reverse-transcribed and the expression of *zirT* and *zirS* was examined by real-time PCR. The fold change in the expression of *zirT* and *zirS* in cultures that were grown in the presence of zinc (M9+Zn) or metal chelators (LB+DTPA/TPEN) relative to their expression in the appropriate unsupplemented media is presented. Expression was normalized using the housekeeping *rpoD* gene as a control. The results represent the average of 3 independent experiments (each included 3–5 replicates) with a standard deviation shown by the error bars.

In order to understand better the regulation the ZirTS pathway, we were interested in identifying different regulatory factors that govern the expression of *zirTS*. Using the Virtual-Footprint program (http://www.prodoric.de/vfp/) [29] we were able to identify a potential binding site for OxyR located 97-bp upstream from the start codon of *zirT*. The OxyR transcription factor is a LysR-type regulator that activates the expression of numerous genes in response to oxidative stress [30]. To test whether OxyR affects *zirTS* expression, *S.* Typhimurium SL1344 *oxyR* mutant strain and a reporter-gene construct harboring a fusion between *zirTS* and a promoterless β-galactosidase gene (*zirTS::lacZ*) were constructed. Since *zirTS::lacZ* was found to be most strongly induced in M9 minimal medium (pH 7.4) in comparison to LB (742±73 and 258±21 M.U., respectively), the expression of this reporter-gene fusion was compared in both strains under these conditions. As demonstrated in Figure 6A, *zirTS::lacZ* expression was found to be about 2.3 fold higher in the Δ*oxyR* background than the wild-type strain (*P*<0.0001), suggesting that OxyR is involved in the regulation of these genes. To further confirm these results we applied a qualitative real-time PCR approach and compared the abundance of *zirT* and *zirS* transcripts in the Δ*oxyR* background vs. the wild-type strain. In agreement with the *lacZ* reporter-gene results, the expression levels of *zirT* and *zirS* were about 1.5 (*P* = 0.0033) and 2.5 (*P*<0.0001) fold, respectively, higher in the Δ*oxyR* background, compared to the wild-type strain (Figure 6B). Together, these results suggest that OxyR plays a role as a negative regulator of the ZirTS pathway.

The induced expression of *zirTS::lacZ* in M9 minimal medium in comparison to LB and the initial identification of ZirS as a *Salmonella* protein, which presented some sequence homology to eukaryotic zinc-binding proteins, prompted us to examine possible effect of different metals ions on the regulation of *zirTS*. To investigate this, we complemented defined M9 medium with different metal salts and examined the expression levels of *zirTS::lacZ* under these conditions. Addition of Mg^2+^, Fe^2+^ or Fe^3+^ ions to the medium did not alter the expression of *zirTS::lacZ*; however, addition of subinhibitory concentrations of Zn^2+^ ions resulted in moderate but statistically significant (*P*<0.0001) reduction of *zirTS::lacZ* expression, in a dose-dependent manner (Figure 6C). These results implied that Zn^2+^ may repress the expression of *zirTS*. If this assumption were true, we expected that addition of metal chelators to LB broth would lead to an increased expression of *zirTS::lacZ*. Indeed, addition of different divalent metal chelators (DTPA, EDDA, and TPEN) resulted in a significant (*P*<0.0001) increase of *zirTS::lacZ* expression. Furthermore, when the presence of these metal chelators was counteracted by the addition of excessive Zn^2+^, induction was prevented. Addition of excessive Fe^2+^ did not prevent *zirTS::lacZ* induction, indicating that the observed repression is zinc-specific (Figure 6D).

In order to further support these results, we implemented a quantitative RT-PCR methodology and compared the abundance of the *zirT* and *zirS* transcripts in minimal medium supplemented with or lacking zinc, as well as in LB in the presence or absence of metal chelators. As illustrated in Figure 6E, the addition of zinc salt to an M9 defined medium, decreased the expression of *zirT* and *zirS* by about 2 and 2.5 fold, respectively (*P*<0.0001). As oppose to that, when the metal chelators DTPA was added to LB broth, a moderate induction of *zirS* expression, by more than 2.6 fold (*P*<0.0001) was observed. Strikingly, when LB was supplemented with the intracellular zinc-specific chelator, TPEN [31], stronger induction by more than 2 and 5 fold, was observed (*P*<0.0001) in the expression of *zirT* and *zirS*, respectively.

Collectively, we concluded from these experiments that zinc significantly contributes to negative regulation of the ZirTS pathway and therefore we named STM1668 and STM1669, zinc regulated secreted protein (*zirS*) and zinc regulated transporter (*zirT*), respectively.

### ZirTS modulates virulence during salmonellosis in the mouse model

To investigate the role of ZirTS in vivo, we used the murine model for systemic salmonellosis and evaluated the survival-time of BALB/c mice infected orally with ∼1×10^6^ cfu of wild-type, Δ*zirS*, and Δ*zirT S*. Typhimurium strains. Surprisingly, the median survival-times of Δ*zirS* and Δ*zirT* strains were 7.5 and 6 days, respectively, while the median survival-time of the wild-type strain was longer (8.5 days), implying the possibility that the Δ*zirS* and Δ*zirT* strains might possess virulent capability higher than the wild-type. However, although a trend was apparent, with a sample size of 8 mice in each group, these differences were not statistically significant (*P*>0.05).

**Figure 7:**
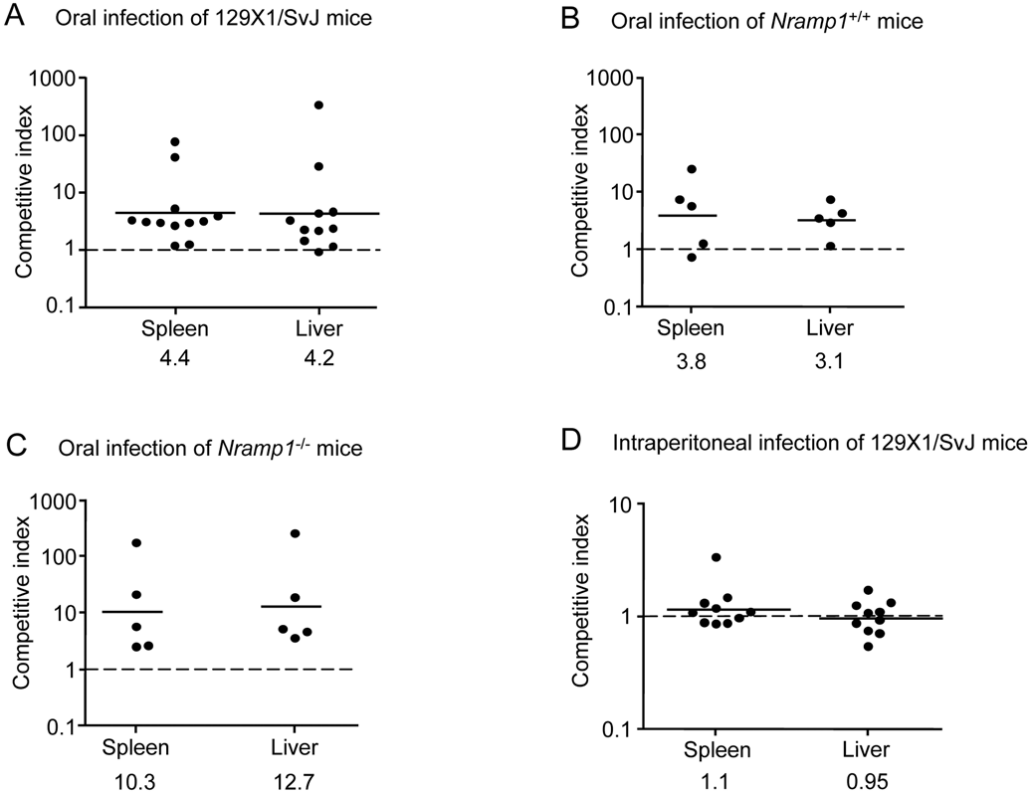
ZirS modulates *Salmonella* virulence during systemic disease in mice. The geometrical means of competitive index values are shown for the spleen and liver of mice that were infected orally (panels A–C) or i.p. (panel D). Six to seven weeks old 129X1/SvJ (panels A and D), *Nramp1* ^+/+^ (panel B), or *Nramp1* ^−/−^ (panel C) female mice were infected with a 1:1 mixed inoculum of marked wild-type SL1344 strain and a Δ*zirS* mutant. For oral infection, mice were inoculated with 1×10^6^ cfu in 0.1 ml of infection buffer (0.1 M HEPES pH 8.0, 0.9% NaCl) and sacrificed after 6 days. For i.p. infection, 2×10^4^ cfu were injected in 0.2 ml PBS and mice were sacrificed after 3 days. Panels A and D represent pooled results from 2 independent experiments.

In many cases, comparing survival time is not sensitive enough to reveal virulence differences, especially when two virulent strains are compared. In contrast, the competitive index (CI) approach [32] is considered to be more sensitive to subtle differences. In these experiments, mice were challenged orally with a mixed inoculum containing equal numbers of wild-type bacteria and a mutant strain carrying an in-frame deletion of *zirS*. Six days post infection (p.i.) mice were sacrificed and the recovered cfu ratio between the mutant and the wild-type strain (i.e. CI value) was evaluated. As the main sites of *Salmonella* replication during systemic infection are the spleen and the liver [33]–[35], the CI geometrical mean was calculated for these sites. In 129X1/SvJ mice, the mean CI was found to be 4.4 and 4.2 for the spleen and liver, respectively (Figure 7A), indicating that the Δ*zirS* mutant strain outcompeted the wild-type strain by more than 4 fold during the infection. Comparable results were also obtained when a Δ*zirT* mutant was competed against the wild-type strain (data not shown). This CI analysis correlates with the single infection results, which suggested a shorter survival-time of mice infected with Δ*zirS* and Δ*zirT* mutant strains in comparison to the wild-type, and together demonstrated a hypervirulent phenotype for Δ*zirS* and Δ*zirT* mutant strains in mice.

*Nramp1* (natural resistance-associated macrophage protein-1; also known as Slc11a1) is a host resistance gene that provides protection against several intracellular pathogens, including *S.* Typhimurium [36]. In order to examine a possible effects of Nramp1 on the observed hypervirulent phenotype, and to further validate these results, we repeated the CI analysis in 129Sv/J (*Nramp1* ^+/+^) and isogenic Nramp1-deficient (*Nramp1* ^−/−^) mouse genetic backgrounds. As can be seen in Figure 7B and C, a similar trend was observed in these mouse strains, with an even more pronounced difference in the *Nramp1* ^−/−^ background. In the latter, the mean CI values were 10.3 and 12.7 in the spleen and the liver respectively, demonstrating a significant overgrowth of the Δ*zirS* mutant in comparison to the wild-type strain.

Next, we examined whether the apparent hypervirulence behavior of the Δ*zirS* mutant is dependent on the route of infection. To test this, 10 129X1/SvJ mice were infected intraperitoneally (i.p.) with approximately 2×10^4^ cfu and sacrificed 3 days p.i.. Intriguingly, as indicated in Figure 7D, when the bacteria were administrated i.p., both strains reached equal numbers and no growth advantage of the Δ*zirS* mutant was observed, suggesting that following i.p. infection, wild-type and the Δ*zirS* mutant are equally virulent. Similar results were obtained during CI infection of a Δ*zirT* mutant versus a wild-type strain (data not shown).

We infer from these experiments that the absence of the *zirS* (or *zirT*) gene leads to hypervirulence of *Salmonella* in vivo, resulting in a significant (3–12 fold, *P*<0.05) overgrowth of the Δ*zirS* strain in systemic sites. Interestingly, the differential growth of the Δ*zirS* mutant was evident only following oral infection but not when the mice were infected i.p.. Since the lack of ZirTS leads to an increased virulence of *Salmonella*, we propose that the ZirTS secretion pathway functions as an antivirulence modulator during systemic disease in mice.

### *zirT* displays a unique expression pattern in vivo

**Figure 8:**
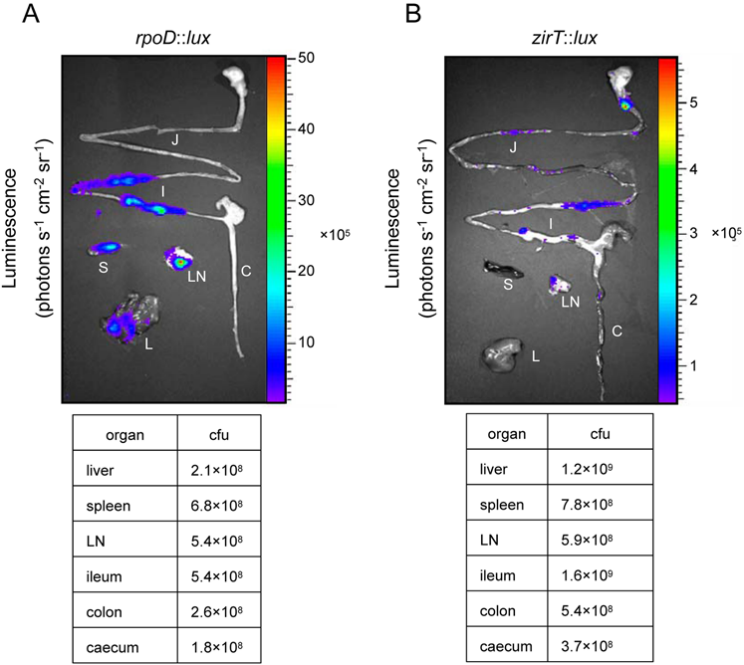
*zirT* is induced and displays a unique expression pattern in vivo. Two groups of female C57BL/6 mice were infected orally with ∼10^7^ cfu of wild-type *S.* Typhimurium expressing *rpoD::lux* (A) or *zirT::lux* (B) reporter strains. At day 3 p.i. mice were sacrificed and their intact GI tracts as well as systemic organs were removed and imaged immediately. The detected bioluminescence signal is shown as pseudocolor images, with variations in color representing light intensity at a given location. The color bar indicates relative signal intensity (as photons s^−1^ cm^−2^ sr^−1^). Different organs are indicated as follow: liver (L); spleen (S); mesenteric lymph nodes (LN); colon (C); ileum (I); and jejunum (J). The experiment was repeated twice (with 9 mice in total for each reporter strain), and representative images are shown. Bacterial load is indicated by a cfu per organ in a table below each image.

The results attributing an antivirulence function to *zirTS* during *Salmonella* infection in mice, prompted us to study the expression pattern of the ZirTS pathway during the course of both acute and persistent infections in the murine model. A reporter-gene fusion between *zirT* and the *luxCDABE* operon was constructed and introduced into wild-type *S.* Typhimurium. As a positive control for in vivo expression, we used a similar construct containing a *Salmonella* sigma 70 (RpoD*)* dependent promoter [37]. Both reporter strains, designated *zirT::lux* and *rpoD::lux*, were used to infect C57BL/6 mice orally. Three days post-infection mice were sacrificed and immediately examined for luciferase activity in the gastrointestinal (GI) tract and systemic sites. Although *zirT::lux* had about two logs lower expression levels in vitro in comparison to *rpoD::lux* (data not shown), induced and distinct expression of *zirT::lux* was observed in vivo. In contrast to *rpoD::lux*, which showed strong expression in systemic sites (liver, spleen, and mesenteric lymph nodes) and the small intestine, *zirT::lux* expression was localized primarily within the small intestine, while at systemic sites, *zirT::lux* expression was low, despite being heavily colonized with *Salmonella* (Figure 8). These results suggest that the in vivo expression of *zirT* is induced mainly throughout the gastrointestinal tract of infected animals rather than at systemic sites. This unique expression pattern is in agreement with the CI experiments that showed a virulence difference between the wild-type and a *zirS* mutant strain following oral, but not i.p., infection. In light of these data, suggesting that *zirT* is not abundantly induced at systemic sites; we propose that by administrating the bacteria i.p., the induction of *zirT* in the GI tract was bypassed and differences between the wild type and the *zirTS* mutants were not noticeable.

**Figure 9:**
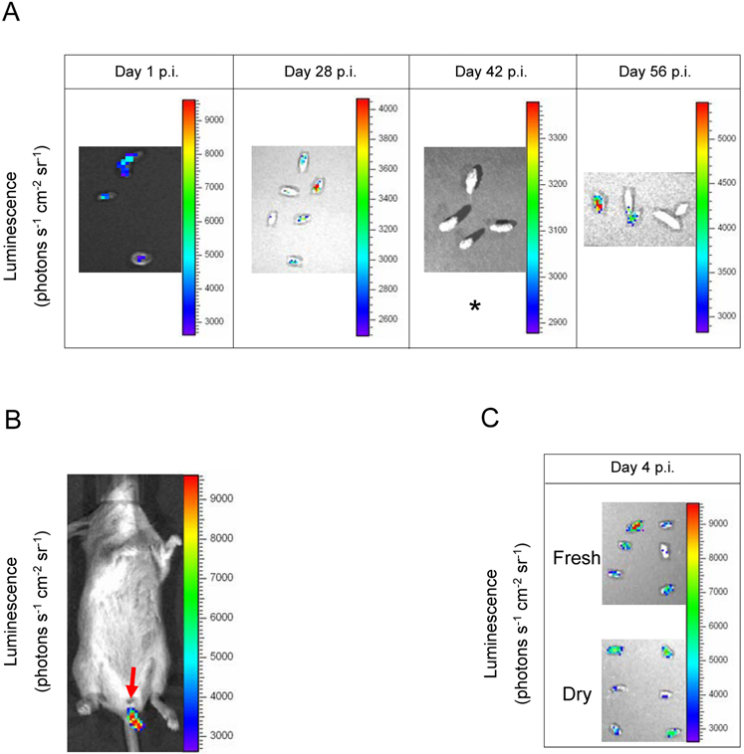
The expression of *zirT* is induced in shed *Salmonella* within fecal pellets during persistent and acute infection. *S.* Typhimurium expressing *zirT::lux* was used to infect 129X1/SvJ and C57BL/6 mice as explained above. (A) Fresh fecal pellets were collected from 129X1/SvJ mice with persistent *Salmonella* infection and imaged immediately. Bioluminescence of fecal pellets is shown for 1, 28, 42, and 56 days p.i.. An asterisk at day 42 p.i. indicates low levels of *Salmonella* shedding in the collected stool as was determined by cfu count. (B) In vivo imaging of 129X1/SvJ mouse, 1 day p.i. The induced expression of *zirT::lux* in the stool is shown. (C) Bioluminescence of fecal pellets from C57BL/6 mice developing an acute *Salmonella* infection is shown for day 4 p.i. Fresh pellets were harvested directly from the animal prior to imaging and dried stools were collected from the cage.

Besides the induction in the small intestine, strong expression of *zirT::lux* was apparent in the fecal pellets from both C57BL/6 (*Nramp*1 negative) mice that developed an acute systemic disease and 129X1/SvJ (*Nramp*1 positive) mice carrying a persistent *Salmonella* infection (Figure 9). Remarkably, the induced expression of *zirT::lux* in *Salmonella* shed within fecal pellets of both mouse strains was evident starting from day one p.i. and lasting at least 8 weeks p.i. in 129X1/SvJ mice during persistent *Salmonella* infection. At 6 weeks p.i., due to low levels of *Salmonella* shedding (as determined by cfu counts), expression of *zirT::lux* was not detected in the feces, but reappeared at 8 weeks p.i., when high numbers of *Salmonella* were shed. These results indicate strong and continuous expression of *zirT* in the feces of shedding mice.

## Discussion

In this study we described the identification and characterization of a *Salmonella*-conserved autonomous and previously unidentified secretion pathway, termed ZirTS. We showed that this novel secretion system consists of an OM protein, ZirT that is expected to adopt a β-barrel structure and the exoprotein ZirS, which is secreted into the extracellular environment in a strictly ZirT-dependent manner.

The ZirTS secretion system was found to share several important features with members of the TPS system and the Int/Inv family. However, despite the mutual similarities, some fundamental differences exist between the ZirTS and the compared systems. Obviously, as opposed to Int/Inv members, ZirTS function as a secretion system comprised of two separate components. Likewise, comparison of ZirTS to TPS systems reveals several hallmarks of TPS, which are absent from ZirTS. The most characteristic feature of the TPS exoproteins is a conserved N-terminal domain, known as the TPS domain. This region has been identified in all of the TpsA proteins characterized thus far and includes highly conserved NPNGI and NPNL motifs. The TPS domain is necessary for secretion and has been proposed to mediate recognition of the exoprotein by the transporter [8], [9], [38]–[40]. An additional feature of TPS is the sequence conservation of TpsB family members, particularly within the C-terminus [41]. Amino acid sequence analysis and comparison of ZirS and ZirT to other known TPS members indicated the lack of these conserved modules in ZirS and ZirT and therefore differentiate ZirTS from the TPS pathway. Based on this we contend that *zirTS* encode a novel secretion pathway in *Salmonella*.

Adapting the currently accepted mechanism of TPS systems, we propose the following model for the *modus operandi* of ZirTS: pre-ZirS is translocated across the IM, via the Sec translocase. Upon translocation, the signal peptide of pre-ZirS is cleaved off by a specific periplasmic signal peptidase and the mature ZirS is released in the periplasmic space. As was shown for other TPS systems, there appears to be no accumulation of periplasmic intermediates [8] and ZirS is expected to transit through the periplasmic space, only briefly. Following Sec-dependent export across the IM, ZirS interacts with ZirT and then is translocated across the OM to the extracellular environment through a hydrophilic β-barrel pore formed by ZirT.

One of many intriguing questions related to ZirTS is its evolutionary origin. A conceivable scenario for the evolution of such a system might be a molecular separation of an ancestral Int/Inv related protein (or an AT) into two distinct functional polypeptides, early in the evolution of *Salmonella.* Other circumstances that might explain the development of ZirTS include a later acquisition of *zirS* into the genome and the “exploitation” of an already existing OM β-barrel porin for its secretion.

Thus far, Int/Inv and TPS systems have been shown to be primarily involved in different virulence traits of Gram-negative pathogens. In contrast, the ZirTS secretion pathway demonstrated an interesting and somewhat surprising antivirulence activity, rather than a virulence determinant, in the murine salmonellosis model. Presently, it is still unknown if the ZirTS pathway is unique to *Salmonella* or related systems exist in other bacteria. A bioinformatic search for non-invasin/intimin ZirT homologs has led to the identification of a predicted 934 amino acid OM protein (SG0602) in *Sodalis glossinidius*, which share 28% identity and 45% similarity with ZirT (E-value 5e-47). The gene localized immediately downstream to SG0602 is expected to encode a 371 amino acid protein (SG0603), with a predicted signal sequence. Interestingly, *S. glossinidius* is an endosymbiont residing intracellularly in tissues of the tsetse flies and utilize for cell invasion a type III secretion system, which is phylogenetically related to the SPI-1 type III secretion system of *Salmonella* [42]. Although at this point, experimental evidence is still needed, *de facto* secretion of SG0603 in a SG0602-dependant manner will indicate a broader distribution of the ZirTS-like pathways among the Enterobacteriaceae.

Despite the fact that ZirS is being secreted into the extracellular environment, in mixed infection experiments, secretion of ZirS from a wild-type strain did not seem to complement the phenotype of a co-infecting *zirS* mutant. This observation may imply that ZirS exerts its effect only topically or has a short in vivo half-life.

Another interesting feature of ZirTS is its location on a horizontally acquired genomic island known as GEI 1664/1678 [17]. The acquisition of genomic islands by horizontal gene transfer enables bacteria to rapidly gain complex functions from other species and are crucial for the interaction of *S.* Typhimurium with eukaryotic host cells. From an evolutionary and ecological standpoint, infections caused by microbial pathogens that have sustained a long-standing association with their hosts are most often self-limiting or go unnoticed. *Salmonella enterica*, as an example of such a pathogen, has maintained a coexistence with vertebrate hosts for millions of years and evolved extremely sophisticated mechanisms to engage vertebrate hosts. When examined at the cellular and molecular levels, this functional interface reveals an impressive array of bacterial determinants designed to manipulate the host immune system, to sense the host environment or to modulate a variety of cellular processes. The overall picture emerging from close examination is perhaps one of balance, self restraint and sophistication rather than one of uncontrolled hostility towards its host [43]. Most studies in bacterial pathogenesis are directed towards finding genes that promote virulence in the host. Nonetheless, recent studies have demonstrated that indeed, a fine balance during host infection is kept due to the function of a subset of *Salmonella* genes known as ‘antivirulence genes' [44]. Deletion mutations of these genes have led to an overgrowth phenotype in the mouse model. Mouslim and colleagues have shown that a PhoPQ regulated gene, *pcgL*, is involved in stimulating the host immune system to prevent bacterial overgrowth in mouse organs [45]. Another example of an antivirulence gene is *sciS*. A *sciS* mutant strain has been shown to display increased intracellular numbers in J774 macrophages and hypervirulence in mice, when administered intragastrically [46]. A null mutation in a Gifsy-2 phage harbored gene, *grvA*, increased virulence as measured by competitive index experiments in mouse spleens and small intestine [47]. Cumulatively, these studies contribute to a recurring theme in pathogenesis emphasizing the importance of pathogens to limit their effects upon the cells they infect in order to achieve a balance with their host. These examples prove that it is possible for an inactivated gene to lead to an increase in bacterial numbers in host tissues. Increased bacterial loads in the murine host would likely lead to more rapid sepsis and toxic shock, thus increasing lethality and decreasing transmission of the pathogen. The hypervirulence of Δ*zirTS* observed in the mouse model during oral infection and its induction in the fecal pellets at an early stage of the infection are consistent with this concept. As with all *Salmonella* species, *S.* Typhimurium is primarily transmitted through the fecal-oral route. Infected animals excrete *Salmonella* in the feces, which will then gain access to an uninfected host, starting a new infection cycle. Supported by induction of *zirT* in the small intestine and its constant induction in feces, we hypothesize that ZirTS play a role as a ‘virulence modulator’ in the early stages of infection. We propose that ZirTS contribute to a host-pathogen balance after the transmission from an infected to naïve host. The role ZirTS play during an early stage of the infection may be in prevention of premature host death and, perhaps, demonstrate at the molecular level, what was understood by MacFarlane Burnet almost 70 years ago that “there is little point in a microorganism destroying its host in a spectacular fashion if this leaves it with no prospect of being ferried to other vulnerable hosts” [48].

## Materials and Methods

### Bacterial strains and in vitro growth conditions

Bacterial strains and plasmids used in this study are listed in Table 1. *S.* Typhimurium SL1344 was used as the wild-type strain, and all mutants used in this study were isogenic derivatives of SL1344. Bacterial liquid cultures were maintained in Luria-Bertani (LB) broth or M9-glucose minimal medium supplemented with 0.0021% (w/v) histidine (since SL1344 is a histidine auxotroph). The appropriate antibiotics were used at the following concentrations: chloramphenicol, 25 µg ml^−1^; kanamycin, 50 µg ml^−1^; ampicillin, 100 µg ml^−1^; and streptomycin, 100 µg ml^−1^. The metal chelators diethylenetriaminepentaacetic acid (DTPA), N,N′-ethylenediaminediacetic acid (EDDA), and NN′N′-tetrakis(2-pyridylmethyl)ethylene diamine (TPEN) were added to LB in the indicated concentrations.

**Table 1:** Bacterial strains and plasmids used in the study.

Strain or plasmid	Genotype and description*	Reference or source
***Salmonella***		
*S.* Typhimurium SL1344	wild-type Sm^r^ *xyl hisG rpsL*	ATCC
NB24	*ushA::res-cat-res*	[57]
OG2006 (*zirS*)	In-frame deletion of STM1668 in SL1344	This study
OG2007 (*zirT*)	STM1669::kan in SL1344	This study
OG2013	SL1344 *oxyR*::kan transduced by P22 from TA4101	This study
TA4101	LT2 *oxyR1* zii-166::Tn5 SGSC1407	SGSC
***E. coli***		
DH5α	F- φ80*lac*ZΔM15 Δ(*lac*ZYA-*arg*F)U169 *deo*R *rec*A1 *end*A1 *hsd*R17(rk^−^, mk^+^) *sup*E44 *thi*-1 *gyr*A96 *rel*A1 λ-	Lab collection
DH5αλ*pir*	For propagation of π-dependent plasmid	Lab collection
SM10 λ*pir*	*thi thr leu tonA lacY supE recA*::RP4-2-Tc::Mu Kmλ*pir*	[51]
TOP10	*mcrA*Δ(*mrr-hsdRMS-mcrBC*) φ80*lacZ* ΔM15 Δ*lacX*74 *deoR recA*1*araD*139 Δ(*ara, leu*) 7697 *galU galK* λ- *rpsL endA*1 *mup*G	Invitrogen
**Plasmids**		
pACYC184	Tc^r^ Cm^r^ cloning vector	NEB
pBR322	Tc^r^ Amp^r^ cloning vector	NEB
pCR-Blunt	Kan^r^ cloning vector	Invitrogen
pCR-Blunt ΙΙ-TOPO	Zeocin^r^ kan^r^ cloning vector	Invitrogen
pCS26	Kan^r^ cloning vector for *luxCDABE* fusion	[37]
pMC1403	Amp^r^ *lacZY* cloning vector	[58]
pOG-PCR-zirT	*zirT* ORF in pCR-Blunt	This study
pOG-PCR-zirTS	Regulatory region of *zirTS* in pCR-Blunt (for *lacZ* fusion)	This study
pOG-PCR-zirT-2	*zirT* regulatory region in pCR-Blunt (for *lux* fusion)	This study
pOG-RzirT-Km	*zirT*::kan in pRE112	
pOG-TzirS	*zirS* in pCR-Blunt ΙΙ-TOPO	This study
pOG-TzirS-2	Δ*zirS* in pCR-Blunt ΙΙ-TOPO	This study
pOG-TzirS-3	Δ*zirS* in pRE112	This study
pOG-TzirT	*zirT* in pCR-Blunt ΙΙ-TOPO	This study
pOG-WSHA	2HA tag in pWSK29	This study
pOG-UzirT	*zirT* region in pUC-19	This study
pOG-UzirT-2	*zirT* ORF in pUC-19	This study
pOG-UzirTS	*zirTS* in pUC-19	This study
pOG-UzirT-Km	*zirT*::kan in pUC-19	This study
pOG-zirS-HA	*zirS*-2HA in pOG-WSHA	This study
pOG-zirT-3	*zirT* ORF in pBR322	This study
pOG-zirT-4	*zirT* ORF in pBR322 Δ Amp^r^	This study
pOG-zirT-HA	*zirT*-2HA in pOG-WSHA	This study
pOG-zirTS-HA	*zirTS*-2HA in pOG-WSHA	This study
pOG-*zirTS::lacZ*	*zirTS::lacZ* in pMC1403	This study
pOG-*zirT::lux*	*zirT::lux* in pCS26	This study
pRE112	Cm^r^ *sacB*1 suicide vector	[49]
pSIG70-16	*rpoD::lux S.* Typhimurium RpoD (σ^70^) responsive synthetic promoter cloned in pCS26	[56]
pWSK29	Amp^r^ low copy number cloning vector	[59]
pUC19	Amp^r^ high-copy number cloning vector	NEB

*: Sm, streptomycin; Cm, chloramphenicol; Kan, kanamycin; Amp, ampicillin.

### Construction of *S*. Typhimurium SL1344 mutant strains

*S.* Typhimurium OG2006 carrying an in-frame deletion (amino acid 9–270) of *zirS,* and OG2007 harboring a Kan cassette in *zirT*, were generated by allelic exchange using the counter-selectable suicide vector pRE112 [49] containing the levansucrase-encoding *sacB* gene [50]. pRE112 based plasmids were transformed into *E. coli* DH5*α*λ*pir* and then electroporated into *E. coli* SM10λ*pir* [51] that was used as the conjugative donor strain to *S*. Typhimurium SL1344. Streptomycin/chloramphenicol-resistance merodiploid colonies were grown for 4 h in LB broth without antibiotic selection, diluted and then plated onto agar containing 1% (w/v) tryptone, 0.5% (w/v) yeast extract, 5% (w/v) sucrose and incubated at 30°C. Sucrose-resistant colonies were selected, and the presence of the constructed mutation was confirmed by PCR. An SL1344 *oxyR* mutant strain was constructed by P22 transduction from TA4101 (*S*. Typhimurium LT2). The growth of these constructed strains was indistinguishable from the parental strain while growing in liquid culture in vitro.

### Generating hemagglutinin tagged proteins and reporter-gene constructs

Primers used in the study are listed in Table 2. Two-hemagglutinin (2HA) tagged version of ZirS was constructed using the primers OG-61 and OG-63. The resulting PCR product containing the intact sequence of *zirT* following by *zirS* (without the stop codon) was cloned into the vector pOG-WSHA after digest with *Sac*I and *Xba*I. The resulting plasmid harbors a C-terminal fusion of the 2HA tag with ZirS (pOG-zirTS-HA). The primers OG-62 and OG-63 were used to amplify a PCR fragment (containing *zirS* and ∼1-kb upstream to *zirS*) that was cloned using *Sac*I and *Xba*I into pOG-WSHA to generate pOG-zirS-HA. To construct a 2HA tagged version of ZirT we used the primers OG-61 and OG-158 to amplify a PCR fragment that was cloned using *Sac*I and *Xba*I into pOG-WSHA resulting in pOG-zirT-HA.

**Table 2:** List of primers used in the study.

Primer	Sequence (5′-3′)
OG-61	GAGCTCGGGCATAATTTCACAGGCGG
OG-62	GAGCTCCAGCAAAATCCGCATTACGG
OG-63	TCTAGATAGTCTTCCTCTGATGGAAATTTC
OG-89	GAATTCGGGCATAATTTCACAGGCGG
OG-92	CCCGGGGATAATAAGATTATGTTTCGAC
OG-158	TCTAGAATTCGCATCAGAGGTTGCGG
OG-186	GTCGAGATAGTGCATTCTTGCCTGCC
OG-187	GGATCCTGTATTGTCTCCTGCTATATCC
OG-212	TTTCCGGGAATGACTATTGC
OG-215	ATCTGGGCTTGGCGTATTAG
OG-216	GATGCCGACATCCGTTATTC
OG-217	TCCCGATATCCTGCTCTGAC
OG-220	GTGAAATGGGCACTGTTGAACTG
OG-221	TTCCAGCAGATAGGTAATGGCTTC
OG-228	GCCAAGCCCAGATTTAACAG
OG-229	AAAGCCATATGCCGGACGC
OG-230	ACGCGCTACAGTCTTATTAATG

A reporter gene construct containing a translational fusion between *zirTS* and a promoterless *lacZ* gene was generated using the primers OG-89 and OG-92. The resulted PCR product was cloned into pCR-Blunt (Invitrogen), digested with *Eco*RI and *Sma*I and subcloned into pMC1403 to generate pOG-*zirTS::lacZ*.

Reporter-gene fusion of *zirT* with the *luxCDABE* operon from *Photorhabdus luminescens* was generated by PCR amplification using the primers OG-186 and OG-187. The resulted product was cloned into pCR-Blunt (Invitrogen), digested with *Xho*I and *Bam*HI and cloned into pSC26 [37] to generate pOG-*zirT::lux*.

### Secretion assay and immunoblotting

To examine the expression and secretion of ZirS and ZirT, culture supernatant was filtered through a 0.2 µm pore-size filter membrane, concentrated by precipitation with 10% (vol/vol) trichloroacetic acid (TCA), and washed with acetone. The secreted protein fraction and the corresponding bacterial cell pellets were resuspended in 1× sodium dodecyl sulfate-polyacrylamide gel electrophoresis (SDS-PAGE) sample buffer and subjected to Western blot analysis using the appropriate primary antibodies: rat monoclonal anti-HA (α-HA; 1∶2,000; Roche Applied Science) or mouse α-DnaK (1∶2,000; Stressgen Biotechnologies). Rabbit polyclonal antibodies against subunit beta of *E. coli* ATP synthase and OmpA were generous gifts from Gabriele Deckers-Hebestreit and Francisco Garcia-del Portillo, respectively, and were both used in a 1∶10,000 dilution. Goat α-rat, mouse, or rabbit immunoglobulin G conjugated to horseradish peroxidase were used as a secondary antibodies (1∶7,500) followed by detection with ECL reagents (Amersham Pharmacia).

### β-galactosidase assays

β-galactosidase assays were performed according to [52]. The assays were performed with 100 µl of culture, and the substrate for β-galactosidase hydrolysis was o-nitrophenyl-β-D-galactopyranoside (ONPG, Sigma). The background expression of the vector (pMC1403) was 2.76±0.5 and 1.21±0.14 Miller Units (M.U.) when cultures were grown in LB and M9 minimal medium, respectively.

### Quantitative real-time PCR analysis

*Salmonella* RNA was extracted from mid-exponential phase cultures using the Qiagen RNAprotect Bacteria Reagent and the RNeasy mini kit according to the manufacture instructions, including an on-column DNase digest using the RNase-free DNase set (Qiagen). The quantity and quality of the extracted RNA were determined by a ND-1000 spectrophotometer (NanoDrop Technologies). To diminish any genomic DNA contamination, RNA was secondly treated with an RNase-free DNase I (Invitrogen). 0.5 µg of DNase I-treated RNA was subjected to a first strand cDNA synthesis using the QuantiTect Reverse Transcription Kit (Qiagen). Real-time PCR reactions were performed in an Applied Biosystems 7500 Fast Real-time PCR System. Each reaction was carried out in a total volume of 10 µl on a 96-well optical reaction plate (Applied Biosystems) containing 5 µl FastStart Universal SYBR Green Master (ROX) mix (Roche Applied Science); 1 µl cDNA; and two gene-specific primers in a final concentration of 0.3 µM each. Real-time cycling conditions were as follows: 50°C for 2 min; 95°C for 10 min; and 40 cycles of 95°C for 15 s, 60°C for 1 min. No-template and no reverse-transcriptase controls were included for each primers set and template. Melting curve analysis verified that each reaction contained a single PCR product. Reported gene expression levels were normalized to transcripts of *rpoD*, a housekeeping gene that serves as an internal control. Gene-specific primers were designed using PRIMER3 software (http://primer3.sourceforge.net/), are listed in Table 2, and correspond to the following genes: *rpoD*, OG-220 and OG-221; *zirT*, OG-216 and OG-217, OG-216 and OG-229; *zirS*, OG-212 and OG-215, OG-228 and OG230.

### Subcellular fractionation

S. Typhimurium SL1344 strains expressing ZirT-HA or ZirTS-HA were grown for 3 h in LB to late log phase (O.D._600_∼1.0). Cells were harvested at 5,000 *g* for 10 minutes at 4°C, and washed with ice-cold phosphate buffered saline (PBS). All the following steps were performed at 4°C. Cells were resuspended in 1 ml of cold lysis buffer [50mM Tris (pH 8.0), 20% (w/v) sucrose, protease inhibitor cocktail (Roche Applied Science), and lysozyme (100 µg/ml)] and incubated on ice for 1 h to generate spheroplasts. MgSO_4_ was added to final a concentration of 20 mM and spheroplasts were collected by centrifugation for 10 min at 5,000 *g*. The supernatant containing the periplasmic fraction was recovered and the pellet containing the cytoplasm and the membranes fractions was resuspended in 1 ml cold sonication buffer [50 mM Tris (pH 8.0), 20 mM MgSO_4_, RNase A (10 µg/ml), DNase I (5 µg/ml), and protease inhibitor cocktail] and lysed by sonication. Unlysed bacteria were removed by low-speed centrifugation at 5,000 *g* for 10 min. The supernatant was recovered and subjected to ultracentrifugation for 1 h at 100,000 *g* (in a TLA 100.3 fixed angle rotor in Beckman TL100 ultracentrifuge) to pellet the membrane fractions. The supernatant represented the cytoplasmic fraction was recovered and the membrane pellet was washed in cold sonication buffer, repelleted for 30 min at 100,000 *g* and resuspended. This fraction represented the total membranes fraction.

### Sucrose density gradient

S. Typhimurium membranes were prepared from 100 ml cultures expressing ZirT-HA that were grown to late log phase (O.D._600_∼1.0) in LB. Cells were harvested at 5,000 *g* for 10 minutes at 4°C, and washed with 20 ml of ice-cold PBS. All steps were performed at 4°C afterwards. Cells were resuspended in 5 ml of cold PBS containing protease inhibitor cocktail and passed twice through a French Pressure cell at 10000 psi. Debris was removed by centrifugation at 5,000 *g* for 10 min and the clarified cell extract was centrifuged for 1 h at 100,000 *g* (30,000 rpm in a SW41Ti rotor; Beckman). Membrane pellets were resuspended in 250 µl of PBS by repeated passage through a syringe equipped with a 25 gauge needle. To separate inner and outer membranes, 200 µl of membranes were layered on top of a discontinuous sucrose gradient composed of 0.5 ml of 60%, 1 ml of 55%, 2.4 ml of 50%, 2.5 ml of 45%, 2.4 ml of 40%, 1.4 ml of 35%, and 0.8 ml of 30% sucrose in PBS (w/v) and centrifuged for 16 hours at 100,000 *g* in a Beckman SW41Ti rotor. Membrane fractions (800 µl) were recovered from the gradient by using a 20 gauge needle from the bottom of the gradient. Fractions aliquots were separated on a SDS-10% Polyacrylamide gel. The presence of OmpA and the β-subunit of ATPase in the different fractions were used as markers for the analysis of the inner and outer membranes using Western-blot with the respective antibodies.

### Mice and *S*. Typhimurium infection

129X1/SvJ female mice were purchased from the Jackson Laboratories. A breeding colony of inbred strain of 129Sv/J (*Nramp1* ^+/+^) mice and isogenic Nramp1-deficient (*Nramp1* ^−/−^) strain have been previously described [53] and are maintained at the University of British Columbia Animal Facility. All the mice were kept in sterilized filter-topped cages and given food and water *ad libitum*. Experiments were carried out under specific-pathogen-free conditions according to the standard animal care guidelines and protocols of the UBC Animal Care Committee and the Canadian Council on Use of Laboratory Animals. For competitive index (CI) infection experiments, 6–7 weeks old female mice were infected orally or i.p. with mixed inoculum containing a marked wild-type strain resistant to chloramphenicol (*ushA*::*cat*, NB24) and a Δ*zirS* or Δ*zirT* mutant strain (OG2006 and OG2007, respectively). For oral infection, mice were administered with 1×10^6^ cfu in 0.1 ml of infection buffer (0.1 M HEPES pH 8.0, 0.9% NaCl) and sacrificed after 6 days. For i.p. infection, 2×10^4^ cfu were injected in 0.2 ml PBS and mice were sacrificed after 3 days. Spleen and liver were homogenized in cold PBS, diluted and plated on LB plates containing streptomycin for determination of total *Salmonella* cfu. Colonies were replica-plated under chloramphenicol selection for enumeration of SL1344 *ushA*::*cat*. The competitive index was calculated as [mutant/wild-type]_output_/[mutant/wild-type]_input_. CI experiments, in which mice were co-infected with the chromosomally marked strain (SL1344 *ushA::cat*) and an unmarked SL1344 strain, or strains harboring mutation in non-virulent genes, demonstrated a CI value of 1, indicating equal virulence capability in mice [54],[55].

### Bioluminescence imaging of *Salmonella* during murine infection

Wild-type *Salmonella* Typhimurium harboring pOG-*zirT::lux* or pSIG70-16, carrying the *lux* operon under an RpoD dependent promoter [56] as an expression control were grown for 16 h in LB+Kan at 37 °C. Female C57BL/6 mice were orally infected with 2×10^7^ cfu of the reporter strains in 0.1 ml of PBS. At 3 days p.i. mice were anaesthetized with 2% isofluorane carried in 2% O_2_ and imaged using IVIS 100 (Xenogen Imaging Technologies). Greyscale reference images taken under low illumination were collected and overlaid with images capturing the emission of photons from the *lux*-expressing bioluminescent *S.* Typhimurium using Living Image Software version 2.50 (Xenogen). To determine the total numbers of colonizing *Salmonella* (cfu), the spleen, liver, MLN, ileum, caecum and colon were homogenized in PBS using a high-speed mixer mill (MM301; Retsch), diluted and spread plated on LB agar supplemented with streptomycin.

### Statistical analysis

Data of the β-galactosidase assays are expressed as mean±standard deviation. The statistical significance between two mean values was calculated by the unpaired *t*-test with two-tailed P value. The statistical significance of the measured mean fold-change by the qRT-PCR was evaluated by *t*-test against hypothetical value of 1 with two-tailed P value. Data of the CI experiments in mice are expressed as geometrical mean. The statistical significance was calculated by the Wilcoxon Signed Rank Test, against theoretical median of 1 with two-tailed P value. *P*<0.05 was considered to be statistically significant.
